# Phenolic Antioxidants in the Adriatic Halophyte *Limbarda crithmoides*: Variation Across Phenological Stages

**DOI:** 10.3390/foods14213718

**Published:** 2025-10-30

**Authors:** Petra Brzović, Sanja Radman, Ivana Generalić Mekinić

**Affiliations:** Department of Food Technology and Biotechnology, Faculty of Chemistry and Technology, University of Split, R. Boškovića 35, HR-21000 Split, Croatia; sanja.radman@ktf-split.hr

**Keywords:** golden samphire, phenophase, phenolics, antioxidant activity, HPLC, chlorogenic acid

## Abstract

Golden samphire [*Limbarda crithmodes* (L.) Dumort], a perennial, edible Mediterranean halophytic specie, is recognised for its richness in biologically active compounds and its associated health benefits. Despite its potential, it remains largely underexplored in Croatia. This study investigates the effect of plant’s phenological stage—vegetative phase (June), pre-flowering (July), and full flowering (August)—on its phenolic profile (total phenolics, flavonoids, and tannins using spectrophotometry, and individual phenolic compounds using ultra-high performance liquid chromatography and antioxidant potential (using three complementary in vitro assays)). Total phenolics, flavonoids and related antioxidant activity progressively increased from June to August. Among the 18 identified phenolic compounds, chlorogenic acid was the most abundant (355 to 455 µg/g), reaching its highest concentration in the August sample, while among flavonoids it was quercetin (32 to 34 µg/g). These findings suggest that golden samphire collected in the late summer flowering stage exhibits the highest phenolic and antioxidant potential, and that chlorogenic acid, likely acting in synergy with other phenolics, may play the key role in the plant’s biological activity, reinforcing its potential for future applications.

## 1. Introduction

The term halophyte has been used in both ecological and physiological contexts, but one of the most widely acceptable definitions is that halophytes are species capable of growing and reproducing in salt-affected habitats where the salinity of the soil water is around 0.5% NaCl [1,2]. They are almost invariably flowering plants and are represented by about 6000 different species of trees, shrubs, forbs, and grasses [1,3,4]. These species show a wide range of variations within certain growing areas and great diversity in plant systematics, biology, and ecology [3,5]. Halophytes are widespread in a variety of habitats, and, according to O’Leary [1], they grow on all continents except Antarctica.

As a result of their wide global distribution and the fact that they originated and evolved mainly in regions with significant salt concentrations in the soil, which were initially coastal regions such as wet seacoast marshes and estuaries, and then migrated into inland saline areas [1,6,7], there is no single taxonomic group, be it a genus or family, that constitutes the majority of halophytic plants [3].

The halophytic flora of the Mediterranean consists of about 1100 plants within several known families, such as Amaranthaceae and Poaceae, including perennial and annual trees and shrubs [4,5]. One of the families represented in the Mediterranean halophytic flora is the Asteraceae (Compositae) family, also known as daisy, sunflower, aster, or composite family, belonging to the largest families of flowering plants with over 1600 genera and 25,000 species found worldwide [8,9].

Golden samphire (*Limbarda crithmoides* (L.) Dumort., synonym *Inula crithmoides*) is a dicotyledonous and perennial species belonging to the genus *Limbarda* sp. (Asteraceae, tribe Inulae Cass., subtribe Inulinae) and one of the most common Mediterranean halophytes [10,11]. The name *Limbarda* originates from a place in France where this species was first described, and *crithmoides* originates from the similarity of the species to the genus *Crithmum*, specifically *Crithmum maritimum* L. [12]. Golden samphire grows in sandy, loamy, or clayey soil pockets on the coast, where it is exposed to sea spray and can thrive in full sun or light shade. However, it can also grow in salt-drying basins, forming dense monospecific stands. This shrub usually grows up to 1 m and flowers from July to August, while its seeds ripen from August to September [10,11,13].

Like sea fennel, the young leaves and shoots of golden samphire are edible, either raw or cooked, but can also be pickled and used in salads and relishes [10,11,13,14]. It has already been reported that the aerial parts of golden samphire are rich in various pharmacologically active compounds, mainly phenolics, such as chlorogenic, syringic, ferulic, and gallic acids, as well as rutin and quercetin and their derivatives. These compounds were previously investigated by Adorisio et al. [14] and Bucchini et al. [15,16] (Italy), El-Sherbeny et al. [17] and Malash et al. [18] (Egypt), Jallali et al. [19,20] (Tunisia), Oliveira et al. [21,22] and Ferreira et al. [23] (Portugal), Aboul-Ela et al. [24] (Lebanon), and Belloum et al. [25] (Algeria). However, to the authors’ knowledge, apart from a limited study employing non-specific methods (e.g., thin-layer chromatography) by Maleš et al. [26], no data are currently available regarding the phenolics of golden samphire from the Croatian Adriatic coast. Due to its richness in these valuable bioactive compounds, various biological activities have been attributed to the plant, such as antioxidant [15,16,19,20,23,27], antiproliferative [14], antibacterial [20], antifungal [15,16,20], antileishmanial [21], anti-inflammatory [16,21], herbicidal [28], and hepatoprotective [18,24] properties. Although several recent studies have investigated phenolics in golden samphire from the Mediterranean [14,15,16,17,18,19,20,21,22,23,25,27,29], there is a lack of research (or only a limited number of studies) addressing changes in phenolic content across different plant phenophases [22] and, more generally, across Adriatic populations [26,30]. Furthermore, there has been no study dealing with the thorough identification and quantification of golden samphire’s individual phenolic constituents. This research, therefore, aimed to gain insight into the chemical composition and biological properties of golden samphire collected from the Adriatic coast at three different plant vegetative stages (in June at the pre-flowering stage, in July at the beginning of flowering, and in August at the full flowering stage) in order to potentially show the influence of the harvest period on plant phytochemistry and related antioxidant properties, with the goal of determining the optimal harvest period to maximise the plant’s bioactive potential.

## 2. Materials and Methods

### 2.1. Plant Material

**Figure 1:**
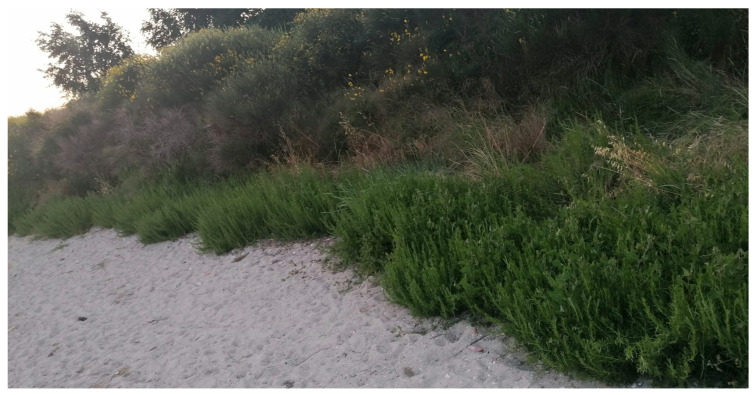
Golden samphire collection site.

The plant material, consisting of the aerial parts of wild-growing golden samphire (*Limbarda crithmoides* (L.) Dumort.) at three different phenological stages (vegetative the phase, the beginning, and full flowering), was collected near the Cetina river estuary (Omiš, Central Dalmatia, Croatia, 43°26′38″ N 16°41′31″ E, Figure 1) in June, July, and August 2023. Approximately 3 kg per sample was collected from several populations within a 10 m^2^ area.

For the chemical analyses, cleaned, fresh plant material was stored at −18 °C and subsequently freeze-dried (FreeZone 2.5, Labconco, Kansas City, MO, USA). Voucher specimens of the plant material were deposited in the herbarium of the Department of Food Technology and Biotechnology at the Faculty of Chemistry and Technology, University of Split (Split, Croatia).

### 2.2. Extraction of Phenolic Compounds

Prior to extraction, freeze-dried plant samples were ground for 1 min using a commercial coffee mill (Model 980, Moulinex, Ecully, France). Extraction was performed as previously reported [29], with slight modifications. The phenolic extracts were obtained by mixing 1 g of freeze-dried plant powders with 10 mL of 50% ethanol in 15 mL conical tubes. The tubes were placed on a mini rotator (Bio RS-24 Mini Rotator, BioSan, Riga, Latvia) and shaken for 1 h at medium speed. After the extraction, the samples were centrifuged for 15 min at 4000 rpm (Centric 322 A, Tehtnica, Železniki, Slovenia), filtered and stored at −18 °C prior to analyses. The extractions for all samples were performed in triplicate.

### 2.3. Spectrophotometric Determination of Phenolics

The obtained extracts were analysed spectrophotometrically for total phenolics (TPC), flavonoids (TFC), and tannins (TTC) using a SPECORD 200 Plus, Edition 2010 spectrophotometer (Analytik Jena AG, Jena, Germany).

The total phenolics were determined using the Folin–Ciocalteu method [31] and the results are expressed as mg gallic acid equivalents per gram of extract (mg GAE/g). The total flavonoids were determined according to the method proposed by Yang et al. [32], and the results are expressed in mg of quercetin equivalents per gram of extract (mg QE/g), while tannins were determined according to the procedure reported by Julkunen-Titto [33], and the results are expressed in mg catechin equivalents per gram of extract (mg CE/g).

### 2.4. Chromatographic Determination of Individual Phenolics

The same extracts used for the total phenolics were used for the determination of individual phenolic compounds. HPLC-DAD (Thermo Scientific UltiMate 3000, Thermo Fisher Scientific Inc., Waltham, MA, USA) was used for the separation and quantification of the individual phenolic compounds. The separation of the compounds was achieved using a Hypersil GOLD™ C18 Selectivity HPLC Column (100 × 3 mm, 3 μm; Thermo Fisher Scientific Inc.) with a constant flow rate of 1.5 mL/min and a temperature of 45 °C. The aqueous mobile phase (mobile phase A) was composed of 0.1% formic acid in water, while the organic phase (mobile phase B) was 90% acetonitrile and 10% mobile phase A. The elution gradient is shown in Table 1.

Peaks were identified by comparing the retention time and absorbance spectra with those of external standards. Calibration curves of the external standards were used for the quantification at 260, 275, 320, and 350 nm. Linearity was verified for all external standards (R^2^ > 0.999), and repeatability was confirmed by triplicate injections (RSD < 10%). The limits of detection (S/N = 3) and quantification (S/N = 10) were estimated from the calibration data. The method’s selectivity was demonstrated by consistent retention times and matching UV–Vis spectra between standards and the sample peaks, as well as by the absence of overlapping peaks in the DAD chromatograms. As three injections were performed for each sample, the results were expressed as mean ± standard deviation (SD) in micrograms of phenolic compound detected per g of dry plant matter (µg/g).

### 2.5. In Vitro Antioxidant Activity Determination

The extracts’ reducing activity was examined using the Ferric Reducing Antioxidant Power (FRAP) assay [34]. The measurements were performed at 593 nm and the obtained results are expressed in µM Fe^2+^/L.

The radical scavenging activity of the samples was tested using the synthetic, stable 2,2-diphenyl-1-picrylhydrazyl radical (DPPH), according to the method reported by Katalinić et al. [31]. The radical inhibition results obtained via recording absorbance at 517 nm are expressed as an IC_50_ (mg/mL), as the sample concentration that provides 50% DPPH radical inhibition.

The Oxygen Radical Absorbance Capacity (ORAC) assay was performed according to the method by Čagalj et al. [35]. The measurements were performed at 485 nm (excitation) and 520 nm (emission), and the results of inhibition of oxidation induced by peroxyl radicals are expressed in µM Trolox equivalents (µM TE).

For the FRAP and DPPH assays, tested extracts were diluted to a concentration of 10 mg/mL and measured by a common spectrophotometer (SPECORD 200 Plus). For the ORAC measurements, extracts were tested at a 1000 times dilution using a microplate reader (Synergy HTX Multi-Mode Reader, BioTek Instruments, Inc., Winooski, VT, USA). All measurements were performed in quintuplicate.

### 2.6. Statistical Analysis

All data are expressed as mean ± SD. For statistical analysis, Statgraphics Centurion Ver. 16.1.11 (StatPoint Technologies, Inc., Warrenton, VA, USA) was used. The least significant differences among the reported results were calculated using one-way analysis of variance (ANOVA) followed by Fisher’s least significant difference test (with a significance level *p* < 0.05).

## 3. Results

### 3.1. Total Phenolic, Flavonoid, and Tannin Contents

**Figure 2:**
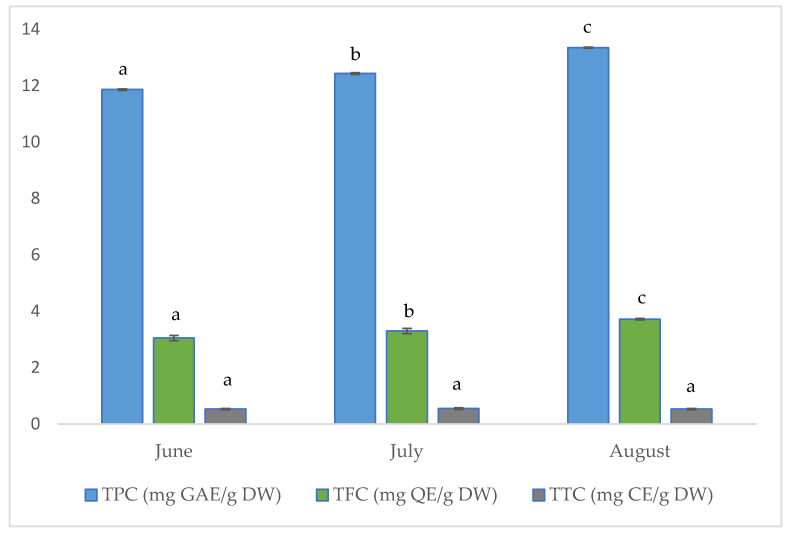
Concentration (per gram of dry weight, DW) of total phenolics (TPC), total flavonoids (TFC), and total tannins (TTC) in samples of golden samphire from different harvest periods. Bars in the same colour (same phenolic group) with different letters (a–c) differ significantly (*p* < 0.05).

The results for total phenolic (TPC), flavonoid (TFC), and tannin (TTC) contents of the investigated aerial parts of golden samphire harvested at different vegetation periods (June, July and August) are shown in Figure 2.

As can be seen in Figure 2, the total phenolic content increased across the collecting periods in the tested golden samphire samples, ranging from 11.86 mg GAE per gram dry weight (DW) for samples harvested in June to 13.35 mg GAE/g for those harvested in August.

There is a lack of previous reports concerning the influence of harvest period and plant phenophase on the chemistry of golden samphire [17], with only one study on plant phenolics [22]. However, several studies have dealt with wildly grown populations of golden samphire, with their focus on the investigation of different plant parts [17,20,23,26,28], collection sites [20,26], used extraction solvents [16,20,23,28], various extraction methods, applied parameters [23], etc.

In their study, El-Sherbeny et al. [17] reported differences in the content of phenolics from various plant parts of golden samphire growing on the Egyptian Mediterranean coast. The authors reported the content of total phenolics in the leaves to be 13.8 mg GAE/g and in the stems to be 9.3 mg GAE/g, which is in accordance with the results of the present study.

Golden samphire was among the five Portuguese halophytic plants that Ferreira et al. [23] analysed for their phenolic content. Due to the differences in the plant material (different plant parts/organs), the solvents used (ethyl acetate, methanol, and water), and the extraction parameters (solid-to-solvent ratio, extraction duration, etc.), the authors reported variations in the phenolic content. However, alcoholic extracts had the highest content of phenolics (4.18 mg GAE/g in the leaves and 2.00 mg GAE/g in the stems), which once again demonstrates the effectiveness of alcohol solvents in the extraction of polar compounds such as phenolics [23]. Similar observations were reported by Bucchini et al. [16] who investigated the influence of different solvents (*n*-hexane, dichloromethane, and methanol) on the extraction of phenolics from Italian golden samphire that was harvested at the flowering stage. The results confirmed that the methanolic extract had the highest phenolic yield (15.52 mg QE/g DW) compared to the other extracts. Jallali et al. [20] investigated the phenolic content and biological activity of the Tunisian golden samphire. The authors investigated different plant parts (root, stem, leaves, and flowers) from samples harvested at three different locations, as well as variations between extracts prepared by solvents with different polarities. Regarding the total phenolic contents in the investigated sample, the concentrations ranged from 15.6 to 100.4 mg GAE/g, depending on the plant part analysed, with the highest concentrations detected in flowers (from 81.2 to 100.4 mg GAE/g). The leaf samples contained smaller amounts of phenolic, ranging from 15.6 to 31.3 mg GAE/g. Oliviera et al. [22] reported variations in phenolic content among golden samphire from different vegetation periods (spring, summer, autumn, and winter), with the largest amount detected in the autumn sample (27.2 mg GAE/g of extract).

The total flavonoid content for the golden samphire samples followed the same trend as the total phenolics content, with flavonoid content increasing as follows: June (3.1 mg QE/g DW) < July (3.3 mg QE/g DW) < August (3.7 mg QE/g DW).

Maleš et al. [26], the authors of the only study on Croatian golden samphire, investigated the flavonoid content of leaves harvested at three different locations using thin-layer chromatography. The authors reported a flavonoid content ranging from 0.2% to 0.43% of the plants’ dry weights. As for the total amount of flavonoids in the Egyptian samples, El-Sherbeny et al. [17] found 2.6 mg CE/g in the stems and 9.3 mg CE/g in leaves, while Ferreira et al. [23] reported 48.1 and 37.4 mg CE/g in Portuguese samples prepared by methanol, respectively. However, when using water as an extraction solvent, the reported values were significantly lower. The content of flavonoids in the study by Jallali et al. [20] ranged from 14.2 to 87.5 mg CE/g, depending on the sample location and plant part analysed. Again, the largest amounts were detected in flowers, while the concentrations detected in leaves ranged from 14.2 to 37.4 mg CE/g. The content of flavonoids in the study by Oliveira et al. [22] ranged from 4.8 to 13.3 mg QE/g of extract.

In this study, no significant differences were found among samples in terms of total tannin content. The detected amount was quite small, and values ranged between 0.53 and 0.55 mg CE/g.

Jallali et al. [20] also investigated the tannins in golden samphire and reported significantly higher levels in their results, which could be primarily affected by the plant samples’ characteristics (harvest location and period), the applied extraction parameters, and the component detection method. Also, their research confirmed a more than two-fold higher content of these phenolics in the leaves (from 7.8 to 13.2 mg CE/g) compared to the stems. El-Sherbeny et al. [17] determined the tannin content in golden samphire stems and reported a concentration of 0.41 mg tannic acid equivalents/g, while Oliveira et al. [22] did not confirm the presence of these compounds in their samples.

Various factors influence the synthesis and accumulation of plant secondary metabolites in halophytes, including phenolic compounds, such as plant phenological stage/harvest period, plant part used, and climatological factors, but also harvest location and soil abiotic factors, etc. [17,19,20,22,26,36,37,38,39,40,41]. Several previous studies investigated the impact of environmental factors, such as collection site and soil salinity, on the content and profile of phenolics in halophytic species. Jallali et al. [20] investigated golden samphire from three collection sites located in different bioclimatic zones (upper humid, upper semi-arid, and upper arid). Despite differences in the phenolic content between the plant parts, no clear effect of locality was observed. Variations in the flavonoid content of Adriatic golden samphire between harvest sites were also reported by Maleš et al. [26]. Although studies on golden samphire remain scarce, some recent studies, summarised and discussed by Blažević et al. [42], on the most widespread halophytic species *Crithmum maritimum* (sea fennel) confirmed high impact of salinity levels on the plant’s physiological adaptations (primarily biomass and growth), as well as on the accumulation of osmolites, antioxidants, and secondary metabolites. Meot-Duros and Magné [43] reported that *C. maritimum* accumulates higher levels of phenolic compounds (mainly chlorogenic acid) when exposed to sandy, nutrient-poor, and saline environments. Similarly, Castillo et al. [44] also confirmed the increased phenolics in *C. maritimum* in higher salt concentrations, highlighting the key role of these compounds in plant adaptation to osmotic stress. In our study, plant material was harvested from a site near the river estuary, where populations were exposed to brackish water, which could potentially result in a lower accumulation of phenolics, as previously suggested in studies on sea fennel. As far as the authors are aware, there is no research comparing the phenolic contents of the aerial parts of golden samphire at different plant growth stages, as is the case with other widespread halophytes, such as sea fennel. Both species are edible halophytic plants native to the Croatian Adriatic coast, and often grow in the same locations and compete with each other. Jallali et al. [19] investigated the phenolics in sea fennel in the aerial parts of the plant, harvested during the vegetative (June) and flowering (August) stages. Similarly to our results, the extracts showed an increase in total phenolics (from 7.16 to 8.27 mg GAE/g) from June to August, while the flavonoid content decreased during the studied period (from 4.77 to 3.45 mg CE/g). Popović et al. [41] analysed the content of secondary metabolites in Croatian sea fennel over a one-year period, and the data obtained showed significant variations among samples, similarly to the study by Generalić Mekinić et al. [45], who analysed plants over the pre-flowering, beginning of flowering, full flowering, fruiting, and seeding periods.

### 3.2. HPLC Analysis of Individual Phenolic Metabolites

**Figure 3:**
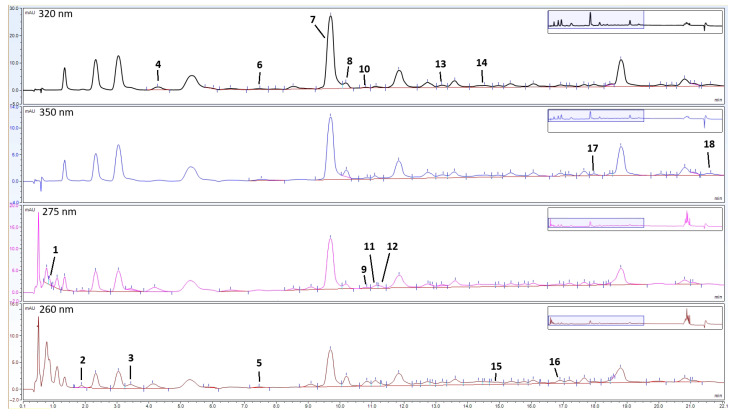
Enlarged chromatograms (from 0 to 22 min) of golden samphire extract from August at 320, 350, 275, and 260 nm. The peak numbers (1–18) correspond to the compounds in Table 2.

The HPLC analyses of the phenolic extracts led to the identification and quantification of 18 compounds in the analysed golden samphire samples, including 12 phenolic acids and 6 flavonoids (Table 2, Figure 3).

The phenolic profile of the individual phenolics in samples is, among other factors, strongly influenced by the plant physiological stage due to different plant needs (biological and ecological functions) in each period [19,26,46,47,48], which is also evident in the reported results.

In most cases, the highest content of detected individual phenolic acids, in agreement with the total phenolics, was found in the extracts obtained during the flowering stage (August sample). This was also the case for chlorogenic acid, which, as shown in Table 2, was the most abundant phenolic compound, with concentrations ranging from 355.14 µg/g in June (vegetative phase) to 455.24 µg/g in August (full flowering). In addition to chlorogenic acid, samples also contained its derivatives, neochlorogenic and cryptochlorogenic acid, respectively. As in the case of chlorogenic acid, the content of neochlorogenic acid increased from July to August, with more than a two-fold increase in its concentration in the flowering sample (26.77 µg/g). Cryptochlorogenic acid content was highest in the sample harvested during the pre-flowering stage (34.72 µg/g), while the samples harvested during the vegetative and flowering stages had similar levels.

Among other phenolic acids, notable amounts in all samples (<10 µg/g) of 4-hydroxycinnamic acid (maximal in July), sinapic acid (maximal in August), and syringic acid (maximal in August) were also detected. Of the detected flavonoids, the highest concentrations were mostly detected in the June sample, and are listed as follows: myricetin (28.79 µg/g), quercetin (34.00 µg/g), epicatechin (18.86 µg/g), and rutin (19.50 µg/g).

The presence of caffeic acid, quercetin, and rutin was previously confirmed in both the leaves and flowers of Croatian populations of golden samphire by Maleš et al. [26]. Jdey et al. [27] analysed the phenolic content of golden samphire extracts from aerial plant parts. Similarly to our studies, the authors found chlorogenic acid in large amounts, albeit at a slightly higher concentration than in our samples (560 µg/g), as well as gallic acid (80 µg/g) and rutin (140 µg/g). Chlorogenic acid is also the main phenolic compound in the halophyte sea fennel, but previous studies on this plant reported the highest concentrations of chlorogenic acids in plant samples from June (vegetative period) [40], which contrasts with the results of this study. Malash et al. [18] confirmed the presence of 3,5-dicaffeoylquinic acid, chlorogenic acid, and quercetin in the aerial parts of golden samphire from Egypt. Aboul-Ela et al. [24] isolated two new quinic acid derivatives, 3,5-di-*O*-caffeoylquinic acid 1-methyl ether and 4,5-di-*O*-caffeoylquinic acid 1-methyl ether, in addition to 1,5-di-*O*-caffeoylquinic acid, from golden samphire samples from Lebanon, while the presence of quercetin in Algerian samples was reported by Bellum et al. [25]. Jallali et al. [20] investigated phenolics in Tunisian golden samphire extracts and detected 1,5-di-*O*-caffeoylquinic acid as the dominant constituent in all plant parts, while their previous study, which only investigated flowers, detected quercetin and its derivative quercimeritrin, and chlorogenic acid and its two derivatives (3-*p*-coumaroyl-5-caffeoylquinic acid and 1,5-di-*O*-caffeoylquinic acid) as major phenolics [19].

### 3.3. Antioxidant Activity

The effect of the golden samphire harvest period on its antioxidant capacity was determined using three different antioxidant assays: FRAP to evaluate the samples’ reducing potential, as well as the DPPH and ORAC methods, which were used to investigate their radical scavenging capacities against, in order, DPPH and peroxyl radicals.

The results, which are shown in Table 3, show a slight increase in antioxidant activity from the first to the last harvest period for all three assays, which is concurrent with the results obtained for the phenolic metabolites considered responsible for the plants’ biological activities [15,16,18,19,20,27].

The FRAP values (at the extract concentration of 10 mg/mL) ranged from 811.51 in the June sample to 1149.43 µM Fe^2+^/L in the August sample. The greatest DPPH radical scavenging activity was, as expected, exhibited by the sample harvested in August, with an IC_50_ value of 22.91 mg/mL extract, while quite similar values were obtained for the other two samples.

The ORAC method was used to test the samples’ inhibition potential against oxidation caused by peroxyl radicals, and the results showed that the extract from the sample harvested in August (full flowering period) had 1.2 times greater activity than the extract obtained from the sample harvested in June (pre-flowering period), and approximately 1.1 times greater activity than the sample harvested in July, when the plant was just beginning to flower.

Golden samphire’s antioxidant potential, as evaluated by DPPH, FRAP, and ORAC assays, can be correlated with the plant’s phenolic content and, more notably, the composition of its individual phenolics, which varied according to the harvest period. As the content of its individual phenolics grew, especially dominant ones, from June (vegetative phase) to August (full flowering phase), so did the antioxidant activity of the plant. This is the case with chlorogenic acid, the most abundant phenolic compound identified in all samples. Chlorogenic acid, an ester of caffeic and (−)-quinic acids, is well-documented in the literature for its strong antioxidant properties [16,48], so it could be suggested that the golden samphire’s antioxidant potential is due to its dominant presence in the plant, especially as it follows the same growth trend during the chosen harvest period. There were other identified phenolic compounds in the extracts, which also participate in the overall antioxidant potential of the samples. For example, neochlorogenic acid concentration also increased from June to August. The extracts also contained flavonoids, such as myricetin, quercetin, and epicatechin, known for their strong radical-scavenging activities [49].

Previous studies have also reported on the free radical scavenging [15,16,19,20,23,27,38,50] and reducing activity [19,20,23,27] of golden samphire phenolic extracts; however, the comparison of results is extremely hard, primarily due to the different procedures in extract preparation, and the methodologies and antioxidants assays used.

Bucchini et al. [15] examined the DPPH scavenging activities of golden samphire callus cultures, which increased with age, and reported the lowest IC_50_ values for 20-day-old callus culture grown in the presence of light. In another study, authors [16] also examined the DPPH radical scavenging activities of different samples, which were obtained using sequential extraction from the aerial parts of Italian golden samphire. In this study, the highest antioxidant potential was detected for methanolic (0.59 mg/mL) and hexanoic (0.57 mg/mL) extracts, and the authors concluded that antiradical activity is linearly related to phenolic content of the extracts.

Jallali et al. [50] investigated the antioxidant activity of golden samphire acetone shoot extracts using the DPPH and FRAP methods and reported that the IC_50_ values differed significantly between the samples from the two collecting sites. Also, compared to the sea fennel extracts that were also investigated, golden samphire had better antiradical activity and lower reducing potential. In a later study [19], the authors used the same methods for the evaluation of the antioxidant activity of the golden samphire flower fractionated extracts, and attributed the good antioxidant activity of the extracts to the presence of chlorogenic acid and its two derivatives, and quercetin. The impact of the presence of 1,5-di-*O*-caffeoylquinic acid on the overall antioxidant activity of the ethyl acetate extracts of golden samphire was reported and discussed by Jallali et al. [20].

Jdey et al. [27] investigated the antiradical activity of golden samphire shoot extracts and reported IC_50_ values against DPPH radical over 220 µg/mL, while the reducing activity of the samples was characterised as low, compared to other plants tested. Ferreira et al. [23] characterised the antioxidant compounds in golden samphire extracts using various methods (among them DPPH and FRAP), and reported the highest activity for the methanolic extracts and better results for the leaf extracts compared to the stems in both cases. El-Sherbeny et al. [17] investigated the DPPH radical scavenging activity of leaf and stem extracts and reported a lower IC_50_ of the leaves.

## 4. Conclusions

This research provides insight into the phenolic composition of the golden samphire plant from the Croatian Adriatic coast, as well as the variations in the composition and properties of the plant depending on the harvesting period. The results indicate a clear dominance of chlorogenic acid, with an increased share of other valuable antioxidant components, such as its derivatives (cryptochlorogenic acid and neochlorogenic acid) and flavonoids such as myricetin, rutin, epicatechin, and quercetin. The share of dominant phenolic acids increased throughout the flowering stages, with the highest concentrations found in the August sample, which likely reflects the different physiological needs of the plant during the investigated growth phases. Notably, the enhanced antioxidant capacity observed in the later harvest stages appears to be attributable to the synergistic effects of elevated phenolic acid levels, especially chlorogenic acid. These findings offer fresh insights into the potential utilisation of golden samphire in food, nutraceutical, and pharmaceutical industries.

## Figures and Tables

**Table 1:** Elution gradient for the separation of the individual phenolic compounds.

t (min)	% B
0	0
5	0
20	18
25	18
30	20
31	100
35	100
35.1	0
40	0

**Table 2:** Concentration of phenolic compounds in golden samphire from different harvest periods.

No.	Phenolic Compound	Rt (min)	Concentration (µg/g)		
			June	July	August
1	Gallic acid	0.86	8.22 ± 0.25 ^a^	8.43 ± 0.16 ^ab^	8.85 ± 0.18 ^b^
2	Protocatechic acid	1.86	3.04 ± 0.11 ^a^	3.32 ± 0.10 ^a^	4.34 ± 0.15 ^b^
3	*p*-Hydroxybenzoic acid	3.40	6.69 ± 0.31 ^a^	7.02 ± 0.11 ^a^	8.84 ± 0.47 ^b^
4	Neochlorogenic acid	4.27	11.71 ± 1.68 ^a^	15.16 ± 0.93 ^a^	26.77 ± 2.04 ^b^
5	Vanillic acid	7.45	6.23 ± 0.37 ^a^	5.37 ± 0.22 ^b^	5.87 ± 0.20 ^ab^
6	Caffeic acid	7.49	6.14 ± 0.65 ^a^	4.72 ± 0.32 ^b^	7.48 ± 0.39 ^c^
7	Chlorogenic acid	9.70	355.14 ± 30.26 ^a^	447.13 ± 8.27 ^b^	455.24 ± 31.08 ^b^
8	Cryptochlorogenic acid	10.18	29.73 ± 2.00 ^a^	34.72 ± 0.39 ^b^	30.02 ± 1.93 ^a^
9	Syringic acid	10.19	14.12 ± 0.58 ^ab^	13.10 ± 0.16 ^a^	17.24 ± 2.80 ^b^
10	4-Hydroxycinnamic acid	10.80	10.48 ± 0.13 ^a^	11.73 ± 0.34 ^b^	10.98 ± 0.06 ^a^
11	Epicatechin	11.12	18.86 ± 1.97 ^a^	18.12 ± 2.17 ^a^	18.24 ± 0.66 ^a^
12	Epigallocatechin-3-gallate	11.27	2.65 ± 1.03 ^a^	5.60 ± 2.78 ^a^	3.57 ± 0.90 ^a^
13	Ferulic acid	13.18	9.04 ± 1.88 ^a^	8.60 ± 0.67 ^a^	10.32 ± 0.74 ^a^
14	Sinapic acid	14.59	10.85 ± 0.42 ^a^	12.23 ± 4.51 ^a^	16.73 ± 1.92 ^a^
15	3-Hydroxycinnamic acid	14.94	5.76 ± 2.27 ^a^	5.22 ± 0.72 ^a^	4.65 ± 0.94 ^a^
16	Rutin	16.90	19.50 ± 8.73 ^a^	18.18 ± 1.60 ^a^	19.41 ± 0.61 ^a^
17	Myricetin	17.95	28.79 ± 1.88 ^a^	27.09 ± 1.31 ^a^	27.34 ± 1.01 ^a^
18	Quercetin	21.60	34.00 ± 0.04 ^a^	32.31 ± 0.14 ^a^	32.48 ± 0.21 ^a^

Rt—retention time. Values with different letters (a–c) within the same row differ significantly (*p* < 0.05).

**Table 3:** Antioxidant activity of the golden samphire samples from different harvest periods.

Sample	DPPH (IC_50_ in mg/mL)	FRAP (µM Fe^2+^/L)	ORAC (µM TE/L)
June	24.74 ± 0.26 ^a^	811.51 ± 11.15 ^a^	35.34 ± 0.26 ^a^
July	24.21 ± 0.09 ^a^	1086.69 ± 6.71 ^b^	38.65 ± 0.12 ^b^
August	22.91 ± 0.11 ^b^	1149.43 ± 2.21 ^c^	42.30 ± 0.71 ^c^

DPPH—2,2-diphenyl-1-picrylhydrazyl radical; FRAP—Ferric Reducing Antioxidant Power; ORAC—Oxygen Radical Absorbance Capacity; TE—Trolox Equivalents. Values with different letters (a–c) within the same column differ significantly (*p* < 0.05).

## Data Availability

The original contributions presented in the study are included in the article, further inquiries can be directed to the corresponding author.

## Abbreviations

The following abbreviations are used in this manuscript:

TPC	Total phenolic content
TFC	Total flavonoid content
TTC	Total tannin content
GAE	Gallic acid equivalents
QE	Quercetin equivalents
CE	Catechin equivalents
HPLC	High-performance liquid chromatography
DAD	Diode array detector
RSD	Relative standard deviation
S/N	Signal-to-noise ratio
FRAP	Ferric reducing antioxidant power
DPPH	2,2-diphenyl-1-picrylhydrazyl radical
ORAC	Oxygen radical absorbance capacity
SD	Standard deviation
ANOVA	Analysis of variance
DW	Dry weight
IC_50_	Concentration providing 50% of inhibition
